# C1q-targeted inhibition of the classical complement pathway prevents injury in a novel mouse model of acute motor axonal neuropathy

**DOI:** 10.1186/s40478-016-0291-x

**Published:** 2016-03-02

**Authors:** Rhona McGonigal, Madeleine E. Cunningham, Denggao Yao, Jennifer A. Barrie, Sethu Sankaranarayanan, Simon N. Fewou, Koichi Furukawa, Ted A. Yednock, Hugh J. Willison

**Affiliations:** Institute of Infection, Immunity & Inflammation, University of Glasgow, 120 University Place, Glasgow, G12 8TA UK; Annexon Biosciences, 280 Utah Ave, Suite 110, South San Francisco, CA 94080 USA; College of Life and Health Sciences, Chubu University, 1200 Matsumoto-cho, Kasugai, Aichi 487-8501 Japan

**Keywords:** C1q, GBS mouse model, Axonal injury, Ganglioside, Autoantibody, Acute motor axonal neuropathy

## Abstract

**Introduction:**

Guillain-Barré syndrome (GBS) is an autoimmune disease that results in acute paralysis through inflammatory attack on peripheral nerves, and currently has limited, non-specific treatment options. The pathogenesis of the acute motor axonal neuropathy (AMAN) variant is mediated by complement-fixing anti-ganglioside antibodies that directly bind and injure the axon at sites of vulnerability such as nodes of Ranvier and nerve terminals. Consequently, the complement cascade is an attractive target to reduce disease severity. Recently, C5 complement component inhibitors that block the formation of the membrane attack complex and subsequent downstream injury have been shown to be efficacious in an *in vivo* anti-GQ1b antibody-mediated mouse model of the GBS variant Miller Fisher syndrome (MFS). However, since gangliosides are widely expressed in neurons and glial cells, injury in this model was not targeted exclusively to the axon and there are currently no pure mouse models for AMAN. Additionally, C5 inhibition does not prevent the production of early complement fragments such as C3a and C3b that can be deleterious *via* their known role in immune cell and macrophage recruitment to sites of neuronal damage.

**Results and Conclusions:**

In this study, we first developed a new *in vivo* transgenic mouse model of AMAN using mice that express complex gangliosides exclusively in neurons, thereby enabling specific targeting of axons with anti-ganglioside antibodies. Secondly, we have evaluated the efficacy of a novel anti-C1q antibody (M1) that blocks initiation of the classical complement cascade, in both the newly developed anti-GM1 antibody-mediated AMAN model and our established MFS model *in vivo*. Anti-C1q monoclonal antibody treatment attenuated complement cascade activation and deposition, reduced immune cell recruitment and axonal injury, in both mouse models of GBS, along with improvement in respiratory function. These results demonstrate that neutralising C1q function attenuates injury with a consequent neuroprotective effect in acute GBS models and promises to be a useful new target for human therapy.

## Introduction

The autoimmune inflammatory neuropathy Guillain-Barré syndrome (GBS) causes rapid onset paralysis, with patients often requiring artificial ventilation, and in severe cases leads to permanent disability through axon loss. Treatment with IVIg and plasmapheresis are beneficial [21], but are non-specific and have unclear mechanisms of action. To develop and test a new generation of therapeutics it is crucial to create a relevant animal model that can be used to both understand the pathophysiology and validate the suitability of potential intervention. GBS is in part mediated by anti-ganglioside antibodies induced by preceding bacterial infections including *Campylobacter jejuni* enteritis [40]. Anti-ganglioside antibodies then target nerve surface gangliosides, glycolipids found extensively in nervous tissue membranes [20]. In particular, the axonal variant of GBS (acute motor axonal neuropathy, AMAN) is strongly associated with circulating anti-GM1 and GD1a ganglioside antibodies [17, 25], which can target and bind to axonal and nodal membranes, whilst the Miller Fisher syndrome (MFS) variant is associated with circulating anti-GQ1b ganglioside antibodies with distinct tissue specificity for cranial nerves [3]. Clinical and experimental evidence suggests the pathogenic mechanisms in GBS include complement fixation by these autoantibodies, leading to classical pathway activation. Complement components have been identified along patient nerve Schwann cell abaxonal membrane in demyelinating GBS [10, 30], and C3d and the terminal membrane attack complex (MAC) pore have been located on the axolemma along the internode and at the node of Ranvier in AMAN [8, 9]. Animal modelling indicates that complement deposition at the node of Ranvier with insertion of the MAC pore will allow the uncontrolled influx of calcium ions, which in turn disrupts ionic homeostasis and initiates calpain cleavage of structural and channel proteins including neurofilament and voltage-gated Na^+^ channels [14, 22, 36]. Terminal complement MAC pore formation is linked to acute injury and dysfunction, but the complement cascade also consists of pro-inflammatory components that can recruit immune cells, which themselves may contribute to pathogenesis. Indeed, macrophages have been found extensively in autopsy tissue [8, 9] and while they participate in clearance of debris to promote recovery, they could also have a role in expanding nervous tissue damage through complement directed, cell-mediated attack. Therefore, the complement cascade has great potential as a target for therapeutic intervention [39].

Inhibition of terminal complement activation products has been tested recently in animal models [12, 13, 15, 22, 27]. In GBS mouse models we have reported that C5 complement component inhibition prevented MAC pore formation and consequent axonal degeneration [12, 13, 15, 22]. Inhibition of C5, however, does not eliminate the production of early complement activation products that induce immune cell recruitment to the site of injury and which could cause further damage or delayed recovery. C1q is the first complement cascade molecule in the classical pathway, and binds pathogenic autoantibodies to initiate the cascade. Therefore its inhibition will prevent downstream activation of only the classical pathway, leaving the alternative and mannose-binding lectin pathways intact to counter bacterial infection [28]. In this report, we specifically examine the role of the classical complement cascade by using a mouse monoclonal antibody that inhibits the function of C1q. A similar antibody was shown to effectively reduce inflammatory demyelinating lesions in an *in vivo* mouse model of the complement-dependent disease neuromyelitis optica [28].

For the current study we have applied a mouse model of the AMAN form of GBS using a newly developed transgenic mouse that solely expresses complex gangliosides neuronally [41], thus allowing us to specifically target and injure axons with an anti-GM1 ganglioside antibody. An additional benefit to this mouse strain is that circulating anti-ganglioside antibody will not be sequestered by other extra-neural plasma membranes which would reduce the bioavailability of the antibodies for binding axonal membranes (Cunningham *et al*., in press). We found that the function-blocking antibody against C1q inhibits pathogenesis in both models of GBS – the MFS model used in our previous study, as well as the new AMAN model described in this report. Our results demonstrate that targeting C1q, the initiating molecule of the classical complement cascade, led to axonal protection, improved function and reduced immune cell recruitment, providing proof of concept for anti-C1q targeted therapies in GBS.

## Materials and methods

### Mice

**Fig. 1:**
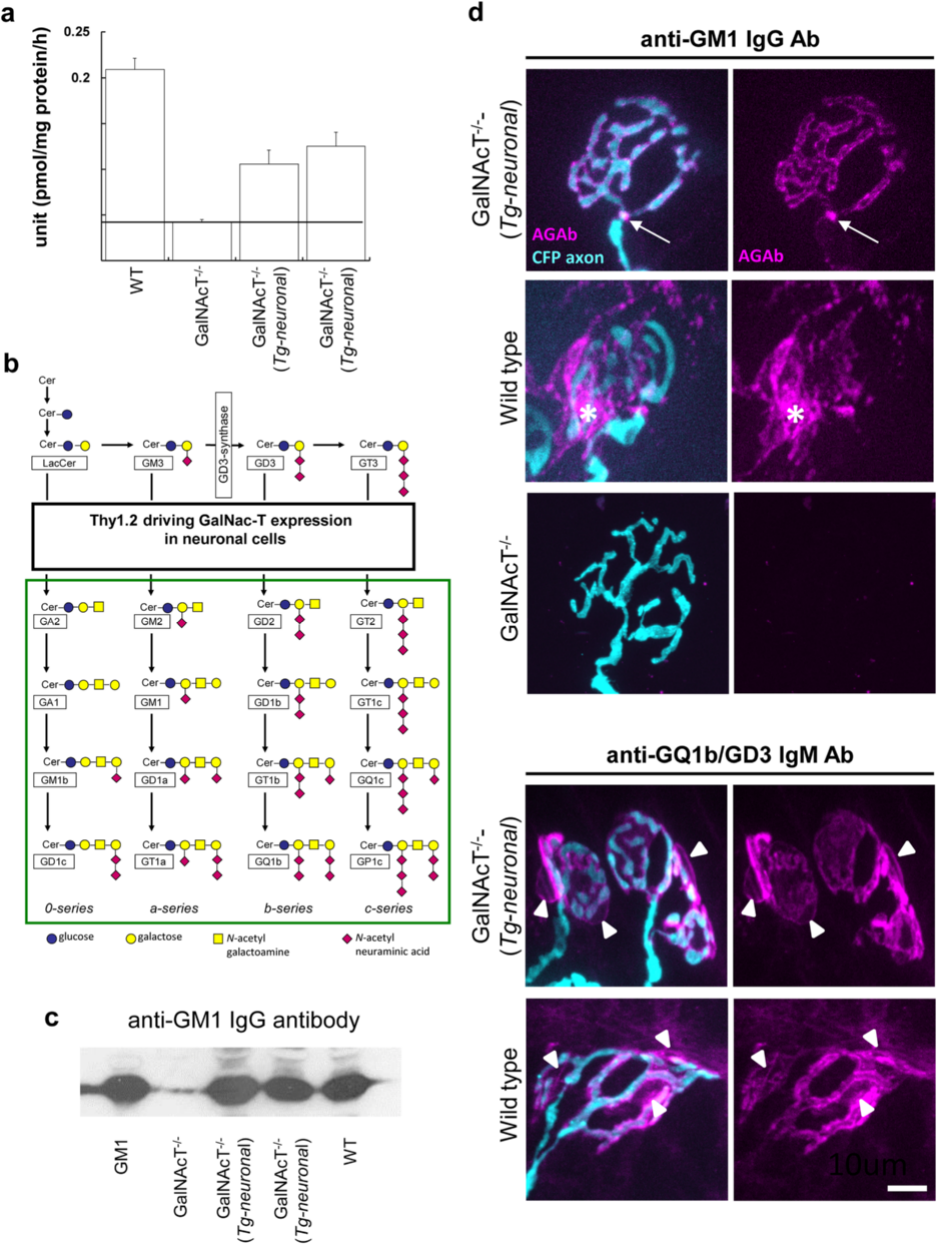
Comparison of ganglioside expression and localisation in wild type and novel GalNAcT^−/−^(Tg-neuronal) mice that exclusively express complex gangliosides in neurons. **a** Enzyme activity assays showed a restoration of GalNAcT activity to ~50 % in 2 different *GalNAcT* ^*−/−*^ *-Tg(neuronal)* lines compared to wild type (*n* = 3/genotype). The dashed line represents the threshold level of activity for the assay therefore *GalNAcT* ^*−/−*^ falls below this and is essentially zero despite minimal value. **b** Ganglioside biosynthetic pathway. The GalNAcT enzyme is necessary for generation of complex gangliosides (surrounded by the green box). **c** Brain extracts from wild type, *GalNAcT* ^*−/−*^ and 2 strains of *GalNAcT* ^*−/−*^ *-Tg(neuronal)* were probed with anti-GM1 IgG antibody, DG2. This antibody bound all genotype extracts except for *GalNAcT* ^*−/−*^ which lack complex gangliosides including GM1. GM1 lipid was printed on the left and provided a positive control for anti-GM1 antibody binding. **d** Axonal binding can be observed in the *GalNAcT* ^*−/−*^ *-Tg(neuronal)* mice treated with anti-GM1 antibody (arrows), while it bound to the terminal kranocyte (asterisk) in wild type mice and was absent in GalNAcT^−/−^ tissue. Anti-GQ1b/GD3 antibody bound similarly in both strains along the axons and on the perisynaptic Schwann cell membranes (arrowheads) that are simple ganglioside GD3 positive. Scale bar = 10 μm

For anti-GQ1b/GD3 antibody mediated injury experiments, Balb/c wild type mice, obtained from Harlan (UK) were used as previously reported. For the anti-GM1 IgG antibody mediated AMAN model *GalNAcT*^*−/−*^*-Tg(neuronal)* transgenic on a C57Bl/6 background were used. For anti-ganglioside antibody binding assessment Balb/c wild type mice, *GalNAcT*^*−/−*^*-Tg(neuronal)* transgenic and wild-type mice both on a C57Bl/6 background were used. All mice were 4 weeks old (12–15 g). Mice had unlimited access to food and water, and housed with a light/dark cycle of 12 h/12 h and constant temperature at 22 °C. *GalNAcT*^*−/−*^*-Tg(neuronal)* mice express the full-length cDNA encoding GalNAcT under the control of the Thy1.2 promoter (restricted to mature neurons) similarly to previously reported mice under NFL promoter activity [41]. GalNAcT cDNA (1655 bp) (provided by Koichi Furukawa) was cloned into the pTSC21K vector (provided by Matthias Eckhardt/Herman van der Putten) for generating Thy1.2–GalNAcT transgenic mice. Transgenic lines and germ-line transmitters were identified by PCR and backcrossed seven generations on a C57BL/6 background. Thy1.2–GalNAcT mice were then interbred with *GalNAcT*^*−/−*^ mice [37] to create 2 lines of *GalNAcT*^*−/−*^*-Tg(neuronal)* mice. Evidence for restoration of GalNAcT enzyme activity in both lines was confirmed by glycosyltransferase activity assays as described previously ([34, 35]; Fig 1a) and evidenced by complex ganglioside synthesis in neural tissues demonstrated by thin layer chromatography overlay (Fig 1c) and immunohistology (Fig 1d) as described previously [41]. For illustrative images, transgenic mice that additionally express cyan fluorescent protein (CFP) in their axons [5] were used. Mice of either sex were killed by CO_2_ inhalation. All experiments using mice were performed in accordance with a licence approved and granted by the United Kingdom Home Office and conformed to University of Glasgow institutional guidelines. Experiments complied with relevant guidelines outlined in the revised Animals (Scientific Procedures) Act 1986.

### Monoclonal antibodies and normal human serum

The IgM anti-GQ1b/GD3 ganglioside monoclonal antibody (mAb), CGM3, was derived from mice inoculated with a GT1a-bearing *C. jejuni* lipooligosaccharide (GQ1b, GD3 and GT1a possess a structural mimic in their terminal trisaccharide to which CGM3 binds) [6]. CGM3 was selected for the MFS model as it induces identical complement-dependent pathogenic effects as human MFS sera *in vitro* [2, 18, 29]. The IgG3 anti-GM1 ganglioside mAb, DG2, was derived from *GalNAcT*^*−/−*^ mice inoculated with the GM1 ganglioside mimicking HS19 lipooligosaccharide [1]. CGM3 and DG2 concentration was measured using quantitative ELISA (Bethyl Laboratories, Texas, USA). The mouse anti-C1q antibody, M1, and a non-specific isotype control IgG1 mAb were provided by Annexon Biosciences (California, USA). Monoclonal antibody M1 binds and neutralises C1q and thereby prevents binding to anti-ganglioside antibody Fc domain and the activation of the classical complement cascade. This antibody is also capable of binding mouse C1q, but since mouse complement is highly regulated by endogenous inhibitors its activation is not involved in injury in our models, as previously shown [39]. Normal human serum (NHS) was collected from a single donor, rapidly frozen and stored in multiple aliquots at −70 °C to preserve complement activity.

### *In vivo* model

Balb/c mice were injected intraperitoneally (i.p.) with 1.5 mg anti-GQ1b/GD3 ganglioside IgM mAb for the MFS model and *GalNAcT*^*−/−*^*-Tg(neuronal)* mice were injected i.p. with 1.5 mg anti-GM1 ganglioside IgG mAb for the AMAN model. After 16 h, mice were injected intravenously (i.v.) into the tail vein with 50 mg/kg anti-C1q neutralising antibody or isotype control mAb to test the effect of anti-C1q inhibition. Several doses were trialled and results indicated that 50 mg/kg was the maximally effective dose, consistent with previous studies [28]. After a further 30 min interval, 0.5 ml 100 % NHS was delivered i.p. The separation of NHS and anti-C1q or control antibody delivery by time and region was performed to avoid immediate inhibition of C1q within the peritoneal cavity. Mice were observed for a further 4–6 h and analysed for whole-body plethysmography, rotarod and grip strength performance (see below). At 6 h, mice were asphyxiated with a rising concentration of CO_2_, and blood, diaphragm and soleus muscles collected for serum and immunohistochemical analysis.

### Plethysmography and behavioural analysis

After training, baseline measurements were collected for all functional and behavioural analysis and were not significantly different between groups for any parameter (see Results). Due to its proximity to the antibody and NHS delivery site, the diaphragm and associated phrenic nerve is the major target structure in this *in vivo* model; therefore whole-body plethysmography (EMMS, Hants, UK) was employed as a non-invasive method for measuring respiratory output and related functional deficits. Interpretation of plethysmography data is complex; even minimal intervention studies (*e.g.* venepuncture) can cause respiratory measurement fluctuation [32]. Nevertheless these recordings are sensitive and provide useful quantification of respiratory failure that could not be achieved by observation, as previously reported [15]. Mice were acclimatised to the equipment and baseline levels recorded before anti-ganglioside antibody injection. Mice were allowed to settle for 30 min, and 5.5 h after NHS delivery, flow-derived parameters of breath frequency and tidal volume were collected from 25 accepted breaths and averaged over this 30 min period. Mice were trained on the accelerating rotarod (30 – 60 rpm for 5 min) prior to treatment. Baseline measurements of the length of time to stay on the rotarod were recorded; subsequently measurements taken 4 h after NHS treatment were calculated as a percentage of this value and compared between groups. Grip strength was performed as described previously [19]. Both fore-limb and all four limb measurements were collected to calculate the hind-limb grip strength (fore-limb subtracted from all four limb), and similarly compared to baseline measurements. Statistical analysis was performed using GraphPad Prism 6 software (GraphPad Software Inc., La Jolla, CA), where significance *p* < 0.05. Student t-tests were applied to compare conditions.

### Immunoassays

#### ELISA

To test for anti-ganglioside antibody reactivity in the mouse sera, ganglioside ELISAs were performed. Immulon-2HB 96 well plates (Thermo Fisher Scientific, MA, USA), were coated with 100 μl at 2 μg/ml of GM1 (Sigma, Aldrich) or GQ1b (Matreya) diluted in methanol, or methanol only for control wells. The average OD reading from control wells was subtracted from all other wells to correct for background. Plates were blocked with 2 % bovine serum albumin (BSA)/PBS solution for 1 h at 4 °C. Block was discarded and mouse serum (1/50 in 0.1 % BSA solution) added overnight at 4 °C. Anti-GM1 and anti-GQ1b monoclonal antibodies DG2 and CGM3, respectively, were used as positive controls and were applied at 50 μg/ml in 0.1 % BSA. Plates were washed 3x in PBS then secondary antibody (goat anti-mouse IgG or IgM-HRP, 1:3000, Sigma Aldrich) was added in 0.1 % BSA solution for 1 h at 4 °C. After PBS washes, OPD substrate solution (30 ml dH2O, 16 ml 0.2 M Na2HP04, 14 ml 0.IM C6HgO7, 1 OPD tablet and 20 μl 30 % H_2_O_2_ added immediately prior to use) was added for 20 min at room temperature (R.T.), followed by stop solution (4 M H_2_SO_4_). Plates were read at 492 nm on a Tecan Sunrise™ automated microplate reader (Tecan Group Ltd, Männedorf, Switzerland) using Magellan™ software.

#### Anti-C1q antibody assay

Serum levels of anti-C1q antibody were measured by ELISA. Black 96 well plates (Costar) were coated with 2 μg/ml of human C1q (Complement Technology) in bicarbonate buffer (pH 9.4) overnight at 4 °C. Next day, the plates were washed with PBS (pH 7.4) (Thermo Scientific) and then blocked with PBS buffer containing 3 % BSA. Anti-C1q antibody standard curve was prepared in the range of 50–0.02 ng/ml in control mouse serum at 1/1,000,000 dilution in dPBS containing 0.3 % BSA and 0.1 % tween. Study serum samples were diluted at 1/1,000,000 in the same buffer. The blocking buffer was removed from the plate. Standards and samples were added at 50 μl per well and incubated shaking at 300 rpm at R.T. for 1 h. Then, 50 μl of alkaline-phosphatase conjugated goat anti-mouse antibody was added to all wells. After an additional hour of incubation at R.T., plates were washed thrice with dPBS containing 0.05 % Tween. Plates were then developed using 100 μl of alkaline phosphatase substrate (Life Technologies). After incubation for 20 min, plates were read using a luminometer. Standards were fit using a 4PL logistic fit and unknowns converted to concentration. Sample anti-C1q antibody levels were corrected for dilution and then plotted.

#### Human C1q assay

Serum levels of human C1q were measured using a sandwich ELISA. Black 96 well plates (Costar) were coated with 1 μg/ml of JL1 antibody (Abcam) in bicarbonate buffer (pH 9.4) overnight at 4 °C. Next day, the plates were washed with dPBS (pH 7.4) and then blocked with dPBS buffer containing 3 % BSA. Human C1q (Complement Technology) standard curve was prepared in the range of 200–0.01 ng/ml in dPBS containing 0.3 % BSA and 0.1 % tween. Study serum samples were prepared in the same buffer at 1/10,000 to 1/30,000. The blocking buffer was removed from the plate. Standards and samples were added at 50 μl per well and incubated shaking at 300 rpm at R.T. for 1 h. Then, 50 μl of alkaline-phosphatase conjugated human-C1q-specific antibody was added to all wells (clone 4A4B11 hybridoma from ATCC HB-8327TM). Plates were incubated overnight with shaking at 4 °C. Next day, plates were washed thrice with dPBS containing 0.05 % Tween and then developed using 100 μl of alkaline phosphatase substrate (Life Technologies). After incubation for 20 min, plates were read using a luminometer. Standards were fit using a 4PL logistic fit and unknowns converted to concentration. Sample C1q levels were corrected for dilution and then plotted.

### Immunostaining

Diaphragms were removed upon termination of the experiment, snap frozen, and stored at −70 °C. Tissue was mounted in OCT mounting medium and longitudinal cryosections collected at 8–15 μm on to coated slides. Sections were stored at −20 °C until use. Immunostaining for complement deposits and axonal integrity at the nerve terminal was performed as previously described [15]. Briefly, nerve terminals, were identified by Alexa Fluor 555 conjugated α-bungarotoxin (α-BTx, 1.3 μg/ml, Molecular Probes). FITC-labelled rabbit anti-C3c (1:300, Dako, UK), FITC conjugated anti-mouse IgG/M (1:300, Southern biotech) or anti-human C5b-9 (1:50, Dako) were applied for 1 h at 4 °C. FITC conjugated goat anti-mouse IgG2a (1:300, Southern biotech) was applied for 1 h at 4 °C to detect C5b-9. For neurofilament staining, sections were incubated for 1 h at 4 °C with Alexa Fluor 555 conjugated α-BTx as above, rinsed, immersed in 100 % ethanol at −20 °C for 20 min, then incubated overnight at 4 °C with rabbit anti-neurofilament heavy antibody (1:750, 1211, Enzo Life sciences, UK) followed by FITC conjugated goat anti-rabbit IgG (1:300; Southern Biotech) for 3 h at 4 °C. All detection antibodies were diluted in phosphate buffered saline (PBS).

For illustrative images, whole mount triangularis sterni (TS) muscle *ex vivo* preparations were used, as described previously [15]. Briefly, the muscle was removed and maintained alive in oxygenated (95 % O_2_/5 % CO_2_) Ringer’s solution 116 mM NaCl, 4.5 mM KCl, 1 mM MgCl2, 2 mM CaCl2, 1 mM NaH2PO4, 23 mM NaHCO3, 11 mM glucose, pH 7.4). Muscle was treated with Alexa Fluor 555 conjugated α-BTx (2 μg/ml) and either 100 μg/ml anti-GM1 IgG3 antibody (DG2) or anti-GQ1b IgM antibody (CGM3) for 2 h at 32 °C, transferred to 4 °C for 30 min and a final 10 min at R.T. before rinsing in Ringer’s. Anti-C1q antibody or control mAb was added to 40 % NHS giving a concentration of 100 μg/ml 10 min before application to the preparations for 1 h at R.T. Tissue was then treated with a combinations of C3c and MAC in Ringer’s medium for 1 h at R.T., followed by fixation with 4 % paraformaldehyde in PBS. Application of 0.1 M glycine for 10 min was performed to quench unreactive aldehyde groups. Antibodies and α-BTx AF-647 were reapplied in PBS overnight at 4 °C. For staining intracellular neurofilament, muscle was fixed in 4 % formaldehyde for 20 min followed by 10 min in 0.1 M glycine and then incubated in a permeabilizing solution containing 0.5 % Triton-X100 in PBS for 30 min at R.T., and rabbit anti-neurofilament (1:200) diluted in permeabilizing solution applied overnight at R.T. Tissue was rinsed in PBS and incubated in the following fluorescently conjugated antibodies diluted 1:300; anti-rabbit IgG-FITC and anti-mouse IgG-TRITC and agitated for 3 h at room temperature in the dark. Tissue was rinsed in PBS and mounted in Citifluor mounting medium (Citifluor Products, UK). For the assessment of anti-ganglioside antibody binding only, tissue was washed and fixed with 4 % paraformaldehyde in PBS immediately after anti-GM1 IgG3 (DG2) and anti-GQ1b IgM (CGM3) antibody incubation. Application of 0.1 M glycine for 10 min was performed to quench unreactive aldehyde groups. Tissue was then incubated overnight at 4 °C with anti-mouse IgG/M-FITC (1:300) in PBS. Tissue was rinsed in PBS and mounted in Citifluor mounting medium (Citifluor Products, UK).

For immune cell staining, 15 μm diaphragm sections were used. Sections were incubated with α-BTx (1.3 μg/ml) for 1 h at 4 °C. To identify CD11b-positive leukocytes, sections were incubated with 4 % PFA for 10 min at 4 °C, washed 3x in PBS then blocked for 30 min in 3 % normal goat serum in PBS at 4 °C. Rat anti-mouse CD11b (MCA711G, Serotec) was applied overnight at 4 °C at a dilution of 1:100. For macrophage and neutrophil staining, sections were fixed in freezing ethanol for 10 min before the blocking step. Rat anti-mouse F4/80 (MCAP497, Serotec) was applied overnight in blocking solution at a dilution of 1:300. Neutrophil marker antibody (NIMP-R14 sc-59338, Santa Cruz Biotechnology) was added at 1:50 overnight in blocking solution. The following day, slides were washed for 3 × 5 min in PBS before FITC-conjugated anti-rat IgG secondary antibodies (Southern Biotech) were applied for 3 h at 4 °C. Vectashield mounting media with DAPI was applied and slides were coverslipped and stored at −20 °C before imaging.

### Image capture and analysis

Digital images were captured using both a Zeiss Pascal confocal laser scanning microscope and a Zeiss Axio Imager Z1 with ApoTome attachment. For quantitative analysis of IgM, C3c, MAC and neurofilament, staining was performed in triplicate for each marker, and quantified as previously described [23]. For each marker 45 images were captured per mouse and at least 100 nerve terminals analysed. A qualitative binary approach was used to determine axonal integrity in the AMAN mouse model as follows: by immunostaining, degenerating neurofilament often appears abnormally fragmented, in contrast to well defined branching pattern seen in normal conditions. Accordingly, though the axons may be clearly disrupted, the immunostaining remained over the α-BTx post-synaptic marker thereby resulting in an “intact” axonal integrity result using the analysis software. To overcome this anomaly, terminals were assessed by eye for a normal/abnormal neurofilament immunostaining conformation and the data presented as a percentage of the total terminals. All studies were observer blinded and statistically analysed using GraphPad Prism 6 software, assuming a significance level when *p* < 0.05. Outliers of antibody intensity measurements were removed *via* ROUT analysis (Q = 1 %). For analysis of non-parametric immunohistological data, Mann–Whitney test was used to compare median values per mouse. Box-and-whisker plots were used to display the spread of all data points from each animal. For immune cell analysis, sections were imaged using consistent settings. Images were taken of the NMJ as identified by α-BTx staining, using a 40x objective. Fifteen images were taken per animal per slide. The number of CD11b or NIMP-R14 positive cells per field of view (FOV) was counted and averaged per animal. All parametric data was compared by student *t*-test.

## Results

### Anti-ganglioside antibody binding in different mouse strains

GalNAc-transferase (GalNAcT) is required for the enzymatic biosynthesis of complex gangliosides, and in wild type mice the gene is widely expressed in neuronal, glial and other tissues. In order to limit ganglioside expression to mature neurons, we generated transgenic mice that express GalNAcT under the Thy1.2 promoter, and crossed these mice with mice lacking the endogenous gene through targeted mutation [37]. As predicted, *GalNAcT*^*−/−*^*-Tg(neuronal)* mice express complex ganglioside exclusively in neurons through expression of the GalNAcT enzyme on the Thy1 promoter (Fig 1b and see Methods) [41]. GalNAcT enzyme activity in whole-brain homogenates was greatly reduced in *GalNAcT*^*−/−*^ mice when compared to WT mice (Fig 1a). *GalNAcT*^*−/−*^*-Tg(neuronal)* lines displayed ~50 % activity compared to WT mice, demonstrating partial rescue of GalNAcT enzyme activity (Fig 1a). To confirm the reintroduction and presence of GM1 in these strains, ganglioside fractions were extracted from brains and assessed by anti-GM1 antibody binding. GM1 was evident in *GalNAcT*^*−/−*^*-Tg(neuronal)* and wild type samples, but not in *GalNAcT*^*−/−*^ extracts, thereby demonstrating that GM1 had been functionally reconstituted in the *GalNAcT*^*−/−*^*-Tg(neuronal)* mice (Fig 1c). To determine the precise tissue localisation of the reconstituted complex gangliosides, whole-mount triangularis sterni nerve-muscle preparations from wild type and *GalNAcT*^*−/−*^*-Tg(neuronal)* mice were incubated with anti-GM1 IgG or anti-GQ1b/GD3 IgM antibody and the binding patterns between genotypes were examined (Fig 1d). Anti-GM1 IgG antibody binding is completely restricted to the axolemma at the motor nerve terminal (delineated by endogenous axonal CFP expression) and the nodes of Ranvier (indicated by arrow) in *GalNAcT*^*−/−*^*-Tg(neuronal)* mice. Wild type nerve-muscle preparations exhibited anti-GM1 antibody binding that overlay the terminal on the membrane of parajunctional fibroblasts termed kranocytes (indicated by asterisk), as has been reported previously [7]. Anti-GM1 IgG3 antibody binding is absent from GalNAcT^−/−^ mice nerve terminals, a finding we have reported previously [7]. These results indicate that anti-GM1 IgG antibody binds exclusively to axons, and not to supporting cells in the *GalNAcT*^*−/−*^*-Tg(neuronal)* mice, thereby providing evidence for *GalNAcT*^*−/−*^*-Tg(neuronal)* mice and anti-GM1 IgG for the development of a pure AMAN model in mice. Conversely, anti-GQ1b/GD3 IgM antibody binds the axons and perisynaptic Schwann cells (indicated by arrowheads) of both mouse strains owing to the retained expression of simple ganglioside GD3 at the latter site (see Fig 1b), and GQ1b on the axon. Therefore, we elected to use anti-GQ1b/GD3 IgM antibody mediated injury in WT mice as the MFS model for assessment of C1q complement inhibition.

### Treatment with anti-C1q neutralising antibody reduced levels of human C1q in mouse serum

Mouse sera were collected at the end of the *in vivo* experiments to confirm anti-ganglioside antibody and anti-C1q antibody levels, and assess the impact on circulating human C1q levels (Table 1). Anti-ganglioside antibody was present at comparable levels in all mice from both MFS and AMAN models, confirming an equal delivery of antibody in both anti-C1q antibody and isotype control antibody treated mice. Anti-C1q antibody levels were in the range of 4000–6000 μg/ml in all treated mice, verifying successful systemic delivery of the C1q neutralising antibody. Human C1q levels in the sera were undetectable in mice treated with anti-C1q antibody in both the MFS (*p* < 0.01) and AMAN models (*p* < 0.001) when compared to isotype control antibody treated mice. Mouse C1q levels were also significantly reduced by anti-C1q antibody treatment, consistent with what is known about the antibody’s cross-species binding (data not shown).

**Table 1:** Serum analysis demonstrates M1 treatment is associated with reduced circulating C1q

	MFS model		AMAN model	
	M1 (*n*=4) control mAb (n=3)		M1 (*n*=5) control mAb (*n*=5)	
AGAb serum levels (μg/ml)	0.26 ± 0.02	0.21 ± 0.04	0.42 ± 0.017	0.43 ± 0.023
M1 antibody serum levels (μg/ml)	5656 ± 518.7***	0.0 ± 0.0	4116 ± 496.4***	0.0 ± 0.0
C1q serum levels (μg/ml)	0.83 ± 0.5**	21.25 ± 3.6	1.175 ± 0.56**	15.08 ± 2.2

All values indicate mean ± SEM, **p<0.01, ***p<0.001 compared to control mAb per model, unpaired students t-test

### Treatment with C1q neutralising antibody protects against neuropathy in a mouse model of MFS

**Fig. 2:**
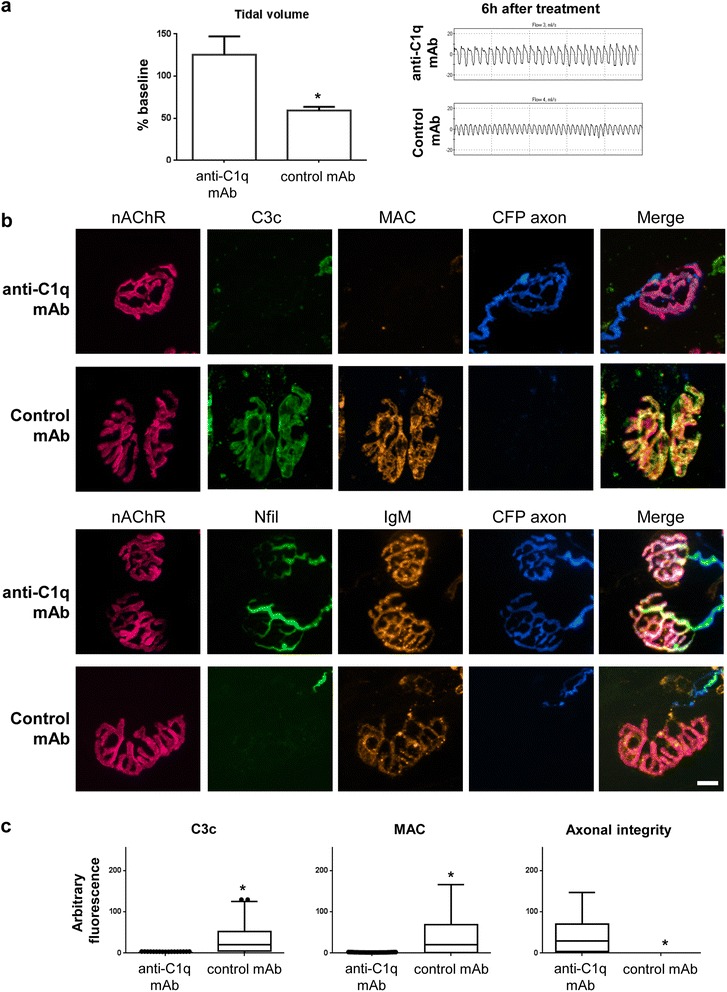
C1q neutralisation attenuates injury in a mouse MFS model. **a** A significant reduction in tidal volume is shown in mice treated with isotype control mAb (*n* = 3) compared to M1 (*n* = 4) antibody (*p* < 0.05). Representative flow-charts from the plethysmography recordings are shown for each treatment group at 6 h post-NHS treatment. Bars represent mean ± SEM. **b** Top panels: Illustrative images show MAC (orange) and C3c (green) deposited at nerve terminals (identified by α-BTx, red; CFP-positive axons, blue) from mice treated with control mAb, while this staining is absent from those treated with anti-C1q antibody. Lower panels: Anti-ganglioside antibody (orange) is present at terminals from both treatment groups, but neurofilament immunostaining (green) is only present at those terminals from mice treated with anti-C1q antibody. **c** The early and end-stage complement products C3c and MAC, respectively, showed significantly greater deposits at control mAb treated mice (*p* < 0.05, *p* < 0.05, respectively) than anti-C1q antibody treated mice (*n* = 3 control mAb, *n* = 4 anti-C1q antibody) nerve terminals. Axonal integrity was a measure of neurofilament immunostaining overlying the endplate. Axonal integrity was significantly more intact at anti-C1q antibody protected mice nerve terminals compared to the control mAb group (*p* < 0.05). Box and whisker plots represent the spread of all data points per condition and significance was based on Mann–Whitney statistical analysis of the median from each animal per treatment. * *p* < 0.05, unpaired student *t*-test (**a**), Mann–Whitney test (**b**). Scale bar = 20 μm. AGAb = anti-ganglioside antibody, nAChR = nicotinic acetylcholine receptor

We previously reported that balb/c wild type mice receiving an i.p. injection of anti-GQ1b/GD3 IgM antibody, followed 16 h later by i.p. administration of normal human serum (NHS, as a source of human complement) develop weakness and difficulty in breathing over the subsequent 6 h [15]. This phenotype reflects intra-diaphragmatic nerve and nerve terminal injury due to the proximity of the diaphragm to the site of anti-ganglioside antibody and complement delivery in the peritoneal cavity. In this study, we replicated this model and found a severe paralytic phenotype, exactly as seen in our previous study [15]. Respiratory function was assessed by whole body plethysmography prior to, and ~5 h after NHS injection. Baseline measurements of tidal volume (anti-C1q antibody = 0.32 ± 0.07 ml, *n* = 4; control mAb = 0.3 ± 0.06 ml, *n* = 4) were not significantly different between the treatment groups. Mice were treated i.v. with either 50 mg/kg anti-C1q antibody or isotype control antibody 30 min prior to the peritoneal injection of NHS. Mice treated with isotype control antibody showed a ~41 % reduction in baseline tidal volume (59.3 ± 4.3 %, *n* = 3, *p* < 0.05). This was a significant decrease in contrast to mice treated with anti-C1q antibody (125 ± 21.9 %, *n* = 4) (Fig 2a), which showed no significant differences in respiratory output from baseline measurements. There was no change in respiratory rate between groups (data not shown). These results demonstrate that treatment with anti-C1q antibody prevent the development of a respiratory function deficit.

We next compared the pathological impact of anti-C1q antibody treatment. The diaphragms from all mice were dissected 6 h post-NHS treatment and nerve terminals assessed for complement deposition and neurofilament immunoreactivity, the presence of the latter signifying axonal integrity. Illustrative examples of this staining is presented in Fig 2b. Fluorescently-labelled α-BTx (magenta) labels the post-synaptic nicotinic ACh receptors and visualises nerve terminals. Additionally, these mice endogenously express CFP in the axons (blue). Early stage complement product C3c (green) and end stage C5b-9b MAC (orange) proteins were identified with immunostaining; staining was present overlying the terminals in mice treated with isotype control mAb, and deposition was prevented in mice treated with anti-C1q antibody (Fig 2b). In mice treated with anti-C1q antibody, where the neuronal fibre remains intact, neurofilament immunostaining (green) shows the axon entry and branching pattern into the neuromuscular endplate, while anti-GQ1b/GD3 IgM antibody (orange) follows the axonal and perisynaptic Schwann cell membrane. The CFP labelling and neurofilament labelling are absent over the α-BTx labelled terminal in mice treated with isotype control mAb, while the anti-GQ1b/GD3 IgM antibody remains. Mice from both treatment groups had similar levels of anti-GQ1b/GD3 IgM antibody in the serum (Table 1) and antibody visualisation at the nerve terminal (Fig 2b, data not shown).

Mice treated with anti-GQ1b/GD3 IgM antibody plus NHS and isotype control mAb, had significantly greater intensity of C3 activation product C3c staining (*p* < 0.05) and terminal complement components C5b-9 that form the MAC pore (*p* < 0.05) (Fig 2c). The group treated with anti-C1q antibody, showed virtually no staining for C3c or C5b-9 complement components at the nerve terminals, while showing robust neurofilament immunostaining at the nerve terminal. In comparison, there was a significant loss in axonal integrity in the isotype control mAb treated mice (*p* < 0.05). These results demonstrate that anti-C1q mAb can prevent neuropathological injury in the mouse model of MFS. While these results are consistent with our previous findings with anti-C5 antibody treatment, the lack of C3 activation as evidenced by a reduction in C3c staining with anti-C1q antibody treatment, demonstrates that the anti-C1q antibody blocks classical complement activation upstream of C3 recruitment and/or cleavage, and thus may potentially also reduce immune cell recruitment and consequently exacerbated axonal pathology.

### Treatment with C1q neutralising antibody protects against neuropathy in a mouse model of AMAN

**Fig. 3:**
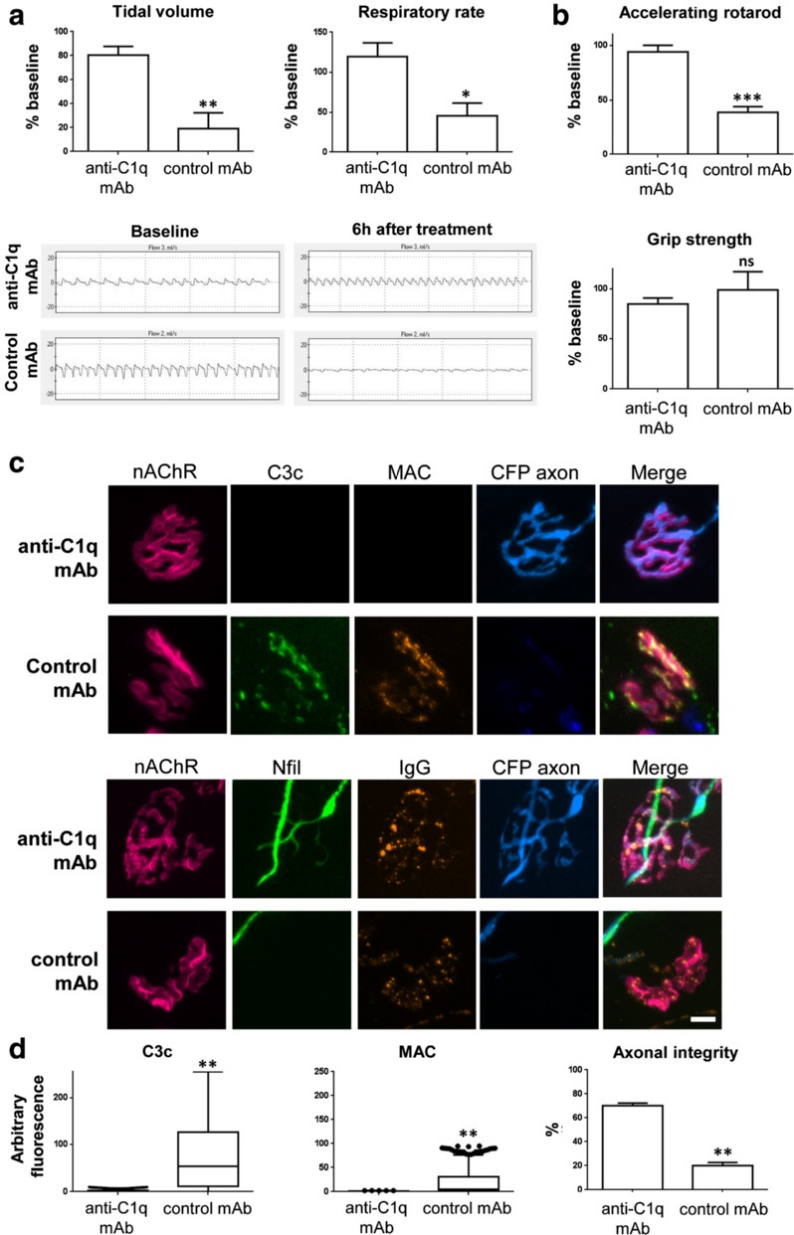
C1q neutralisation attenuates injury in a novel mouse model of AMAN. **a** Tidal volume and respiratory rate decreased compared to baseline measurements in both treatment groups. Tidal volume and respiratory rate reached a significant reduction in mice treated with isotype control mAb (*p* < 0.01, *p* < 0.05, respectively) compared to anti-C1q antibody (*n* = 5/group). Representative flow-charts from the plethysmography recordings are shown for each treatment group at baseline and 6 h post-NHS treatment. **b** Behavioural tests showed isotype control mAb treated mice (*n* = 5) spent significantly less time on the accelerating rotarod than did mice treated with anti-C1q antibody (*p* < 0.001). There was no significant difference in grip strength between groups. **c** Top panels: Illustrative images show MAC (orange) and C3c (green) deposited at nerve terminals (nAChR’s identified by α-BTx, red; CFP-positive axons, blue) from mice treated with control mAb, while this staining is absent from those treated with anti-C1q antibody. Lower panels: Anti-ganglioside antibody (orange) is present at terminals from both treatment groups, but neurofilament immunostaining (green) is only present at those terminals from mice treated with anti-C1q antibody. **d** The diaphragm nerve terminals were identified by fluorescently labelled α-BTx. Nerve terminals were immunohistochemically assessed for complement deposits and axonal integrity. The early and end-stage complement products C3c and MAC, respectively, showed significantly greater deposits at control mAb treated mice than anti-C1q antibody treated mice nerve terminals (*p* < 0.01). Axonal integrity was a measure of percentage “normal” neurofilament immunostaining overlying the endplate. Axonal integrity was significantly more intact at anti-C1q antibody protected mice nerve terminals compared to the isotype control mAb group. Box and whisker plots represent the spread of all data points and significance was based on Mann–Whitney statistical analysis of the median from each animal per treatment. * *p* < 0.05; ** *p* < 0.01; *** *p* < 0.001, unpaired student *t*-test (**a**,**b**), Mann–Whitney test (**c**). Scale bar = 20 μm. AGAb = anti-ganglioside antibody, nAChR = nicotinic acetylcholine receptor

Similar to the MFS injury paradigm, a previously unreported mouse model of AMAN was developed by performing i.p. injections of anti-GM1 IgG antibody into *GalNAcT*^*−/−*^*-Tg(neuronal)* mice to exclusively target axonal membrane. Mice treated with anti-GM1 IgG antibody, NHS and control mAb, developed a ‘wasp-like’ abdominal phenotype with clearly visible difficulty in breathing during the 6 h prior to termination of the experiment. Baseline respiratory measurements from whole-body plethysmography recordings showed no significant differences between treatment groups for tidal volume (anti-C1q antibody = 0.16 ± 0.02 ml, *n* = 5; control mAb = 0.19 ± 0.02 ml, *n* = 5) or respiratory rate (anti-C1q antibody = 277.6 ± 35 breaths/min, *n* = 5; control mAb = 318.3 ± 30.8 breaths/min, *n* = 5). At 16 h following anti-GM1 antibody delivery, 50 mg/kg anti-C1q neutralising antibody or control monoclonal antibody was administered intravenously, followed by i.p. injection of NHS 30 min later. Respiratory function was then monitored by whole-body plethysmography at 6 h post-NHS. There was a significant reduction in both tidal volume (19.1 ± 13.25 %, *n* = 5) and respiratory rate (45.7 ± 15.87 %, *n* = 5) compared to baseline in isotype control antibody treated mice (Fig 3a). In comparison, mice treated with anti-C1q antibody had significantly greater tidal volume (80.44 ± 7.3 %, *n* = 5, *p* < 0.01) and respiratory rate (119.5 ± 17.2 %, *n* = 5, *p* < 0.05) compared to isotype control antibody treated mice; these values did not significantly differ from baseline. Representative respiratory traces are shown highlighting the changes in breathing rate at baseline and 6 h after treatment with anti-C1q antibody or isotype control antibody (Fig 3a).

Behavioural analysis was performed to test for any impairment in motor activity of the mice. Mice treated with isotype control mAb endured significantly less time on the accelerating rotarod compared with anti-C1q antibody treated mice (anti-C1q antibody = 94 ± 6 s *vs.* control mAb = 38.75 ± 5.2 s; *p* < 0.001, Fig 3b), but grip strength did not change between groups. Poor performance on the rotarod is attributed to impaired respiratory reserve as recorded using whole-body plethysmography, rather than impairment of grip strength in the limbs which did not change. This conclusion is corroborated by the identification of anti-GM1 antibody binding overlying nerve terminals of the soleus leg muscle from all mice in the absence of complement activation products, indicating undetectable complement mediated injury to leg nerve terminals (data not shown). Within the time-frame of our experiment, the complement components in NHS needed to drive complement activation/MAC formation directly injure the diaphragm but do not become sufficiently activated in the leg muscles to contribute to a motor functional deficit.

We assessed complement deposition and neurofilament immunoreactivity in nerve terminals in the diaphragm from all mice 6 h post-NHS treatment (Fig 3c), as described above. Mice from both treatment groups had comparable anti-GM1 IgG antibody serum concentration (Table 1) and deposits at the nerve terminals (Fig 3c, data not shown). *GalNAcT*^*−/−*^*-Tg(neuronal)* mice treated with anti-GM1 IgG antibody plus NHS and control mAb, showed significant staining for the early complement product C3c (*p* < 0.05) and terminal complement components C5b-9 (*p* < 0.05) compared to mice treated with anti-C1q antibody, where deposits were undetectable (Fig 3c). In this model, neurofilament immunostaining overlying the terminal was not absent as was often the case in our MFS model, but instead appeared fragmented compared to the normal distinct terminal branching pattern. As such, the quantitative results showed no change in axonal integrity between treatment groups. Instead, we performed qualitative analysis and observed the number of terminals with normal *versus* abnormal fragmented neurofilament immunostaining as a measure of axonal integrity. Indeed, axonal integrity was preserved at the nerve terminals of mice treated with anti-C1q antibody compared to the disrupted neurofilament staining in isotype control antibody treated mice (*p* < 0.01). MAC signal was also less pronounced in this model than in the MFS paradigm and could underlie the less severe neurofilament disruption due to a more incomplete lesion at this time-point. Together, these behavioural and immunohistological data indicate that neuropathy is evident in this mouse model of AMAN and can be rescued by blockade with an anti-C1q antibody.

### Immune cell infiltration is impaired by C1q inhibition in a model of GBS

**Fig. 4:**
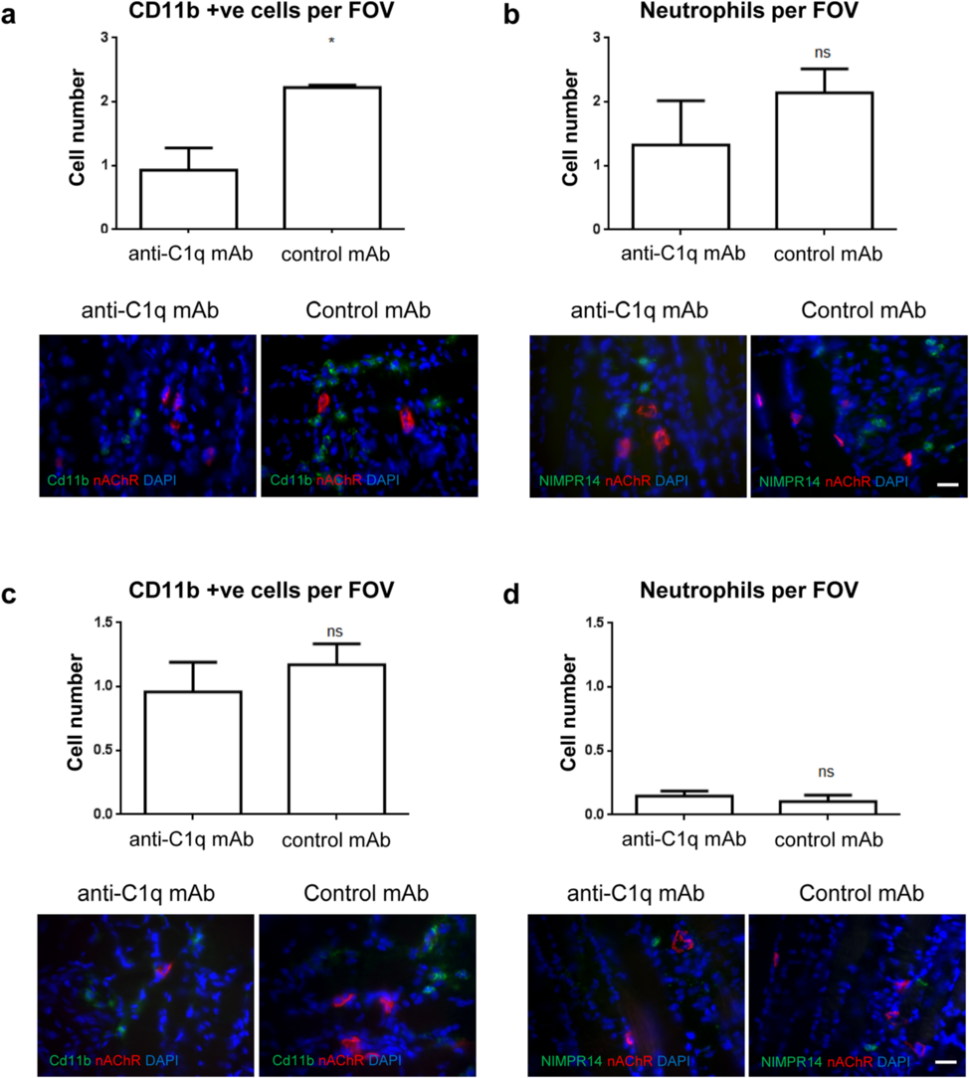
Immune cell infiltration in acute MFS and AMAN models. **a** In the MFS model of disease, there was significantly less CD11b positive cells per field of view (FOV) within the proximity of the NMJ in animals who were treated with anti-C1q antibody. **b** No significance in NIMP-R positive cells existed between the two groups, despite a trend towards lower numbers in the anti-C1q antibody treated group. **c** In the AMAN model of disease, there was no significant difference between anti-C1q antibody treated and control mAb-treated animals in either CD11b or (**d**) NIMP-R14 positive cells. Neutrophils appeared to be absent within the proximity of the NMJ in this model. Bars represent mean ± SEM, *p < 0.05, unpaired student *t*-test. Scale bars = 20 μm. nAChR = nicotinic acetylcholine receptor

Complement pathway products can recruit immune cells and indeed immune cell infiltration has been observed in GBS patient nerve pathology. To investigate immune cell infiltration in an acute model of GBS and any modulation by C1q neutralisation, diaphragm sections from each model were examined for infiltrating cells around the nerve terminals using several markers associated with early and late stage inflammatory cells. In the MFS model, the general leukocyte marker, CD11b, labelled significantly more cells surrounding the nerve terminal in mice treated with isotype control mAb than anti-C1q antibody (Fig 4a, *p* < 0.05). There was a trend toward a significant reduction in neutrophil cell number in anti-C1q antibody treated tissue, but this did not reach significance (Fig 4b). The number of CD11b positive cells (Fig 4c) or neutrophils (Fig 4d) was not significantly different between treatment groups in the AMAN model. Comparatively, the number of cells was lower in the AMAN model compared to the MFS model. Macrophages were not observed in either model or treatment groups, as would be expected over this short duration time course (data not shown). These results demonstrate early signs of leukocyte and neutrophil infiltration in both mouse models of GBS, with a trend for a reduction following treatment with anti-C1q antibody in the MFS model of GBS.

## Discussion and conclusions 

This study presents two major developments in the field of GBS modelling and immunotherapy. Firstly, we report the establishment of a pure, readily reproducible and temporally precise model of AMAN in mice. Using newly developed transgenic mice that selectively express complex gangliosides only in their axonal membranes [41], we are able to exclusively target axons *in vivo* with anti-GM1 ganglioside antibody and a source of complement, resulting in the axonopathic phenotype described herein. The importance of creating an AMAN model lies in understanding the mechanisms underlying the distinct features of axonal and demyelinating GBS variants, and tailoring therapeutic approaches accordingly. Secondly, we report the successful attenuation of clinical and pathological disease in the acute AMAN and MFS models of GBS by targeting the initiating protein of the complement cascade, C1q. This is an as yet untargeted stage of the complement pathway in GBS models and may have advantages over terminal component complement inhibitors. Improved and specific pathway-targeted therapies are greatly needed in GBS, where no new treatments have been approved since the introduction of intravenous immunoglobulin and plasma exchange over 20 years ago.

GM1 antibodies are strongly associated with the axonal variant of GBS, AMAN [17, 25]. It is unclear why motor axons are specifically targeted in AMAN as GM1 is also present in myelin, and in sensory axons [24]. The boundary of this dichotomisation is the subject of considerable on-going clinical interest [38]. By developing a mouse in which complex gangliosides are exclusively expressed in neurons, it is possible to target this site and study the mechanisms that purely reflect axonal injury. Conversely, we are similarly able to study mice in which GM1 expression is restricted to Schwann cell membranes [41]. Here, we report that GM1 ganglioside was successfully reconstituted in brain extracts from *GalNAcT*^*−/−*^*-Tg(neuronal)* mice compared to *GalNAcT*^*−/−*^ mice. Further, we proved by topical immunostaining of the triangularis sterni nerve-muscle preparations that GM1 was only detectable on axonal membranes, which is critical to developing a pure AMAN model. This represents an important step towards evaluating novel therapeutics in AMAN compared to using wild type mice for several reasons. By expressing GM1 in neurons, we can be certain that the early pathology we observe is solely due to direct axonal injury. As GM1 is present on the kranocyte, a parajunctional fibroblast that caps the motor nerve terminal in rodents [4] and in Schwann cell membranes, binding and injury to these sites may confound rigorous interpretation of data, in comparison with wild type mice. An additional major limitation to modelling AMAN in wild type mice is the extensive sequestration of anti-ganglioside antibodies by other extra-neural plasma membranes that limits the bioavailability of the antibodies for targeting axonal membranes (Cunningham *et al.*, Brain in press). This sequestration of antibody by extra-neural cells may in part account for the poor axonal staining visualised with the DG2 anti-GM1 antibodiesin wild-type mice.

In our previously reported acute mouse model of the MFS variant of GBS, administration of anti-GQ1b/GD3 ganglioside antibody and a source of complement caused a respiratory paralytic phenotype in wild type mice [15]. Whilst we have never previously been able to reproducibly induce a similar phenotype with anti-GM1 antibody in wild type mice (preferring instead to use anti-GD1a antibody in GD3 synthase−/− mice that overexpress GD1a [22]), we have here recapitulated this injury paradigm using *GalNAcT*^*−/−*^*-Tg(neuronal)* mice receiving an injection of anti-GM1 ganglioside antibody to produce an entirely original AMAN model. The clinical and morphological phenotypes were successfully reproduced in this model: mice developed weakness, respiratory dysfunction and associated complement deposition and pathology in diaphragm nerve terminals. The new opportunities provided by this novel model are profound - it is now possible to directly and specifically target the axolemma at nerve terminals and the nodes of Ranvier, study associated nodal pathology, and determine the downstream consequences on function and axon fate, currently a major area in GBS clinical research [38]. This model can now be adapted and extended over time to allow the development of the GBS pathological features and motor deficits that are observed in patients, subsequently allowing the effective stages of therapeutic intervention to be scrutinised.

Complement activation on axolemmal membranes has been associated with AMAN in human autopsy studies and animal modelling [8, 9, 22, 36] and thus presents a promising target for therapy. Halstead *et al.* (2008)*,* established an MFS mouse model and used it to show that inhibition of the terminal MAC pore formation using an anti-C5 monoclonal antibody could abrogate pathophysiology [15]. A similar model, focussed on nodal pathology, was also investigated by us with a similarly protective outcome [22]. Using the current *in vivo* AMAN model with anti-GM1 antibody we here report that blockade of the classical complement pathway at the initiating stage using the anti-C1q monoclonal antibody M1 [28] similarly attenuates dysfunction and pathology. Anti-C1q antibody directly targets C1q to inhibit classical complement pathway activation and to prevent MAC pore formation and calcium influx, the latter step being the target of anti-C5 antibody. C1q neutralisation may be beneficial over C5 inhibition in terms of infectious risk, as the mannose-binding lectin pathway is still functional and the host should therefore retain a defence system against bacterial infections. Additionally, blocking C1q will eliminate the generation of anaphylotoxins C3a and C5a, which cause effector cell chemotaxis, binding and degranulation, with the potential for further downstream injury [42]. The safety of blocking C1q in human studies with a therapeutic antibody has yet to be determined. In this hyper-acute model, we provide the evidence that anti-C1q antibody can attenuate complement-mediated injury by applying the anti-C1q antibody prior to addition of an active complement source to the mouse. In future studies in which the model is prolonged, studying the efficacy of C1q neutralization after disease onset will be of interest as this better represents human clinical situations.

Our acute GBS disease models are complement-mediated and we know that dysfunction correlates with MAC pore formation [12, 14, 15]. However other steps within the complement cascade could play a role in injury. Here we studied effector cell recruitment at the diaphragm nerve terminals and any effect of early complement component blockade. Complement activation has the capacity to recruit immune cells to injured peripheral nerves [31], and it is known that neutrophils and macrophages can infiltrate the injured nerve within two days of injury [11]. We report an increase in CD11b positive cells and neutrophils in our MFS model, which was attenuated by anti-C1q antibody treatment. This finding demonstrates that blocking C1q can successfully reduce immune cell infiltration. There was no change in immune cell number in our AMAN mouse model, suggesting immune cells are not involved in the pathogenesis reported over this short time scale as modelled in this paradigm. Lack of immune cell invasion may be attributed to the lower overall level of complement activation observed and production of subsequent soluble signals. The acute duration of the paradigm may also limit immune cell recruitment into the terminals or nodes of Ranvier; it will be of interest to assess their role in extended models in the future. Macrophage infiltration into the periaxonal space of nerves and surrounding degenerating axons has been observed in autopsy tissue from AMAN patients [8, 9]. A rabbit model of AMAN suggested this recruitment of macrophages into the nerve begins at the acute progressive phase (~2 days) of disease but did not yet correspond with sites of complement deposition [36]. Instead macrophage invasion was significantly more frequent at the early recovery phase (~2 weeks), suggesting that these cells are involved in clearance of degenerating fibres rather than injury. At such an acute time-point in our models we did not observe any macrophage infiltration. Further study in an extended model will be necessary to determine which immune cells invade the site and their impact on injury and recovery. Extending the model to several days will also be informative for testing the long-term prognosis and suitability of different therapeutic targets. For example it is known that C1q plays a role in neurite outgrowth and regeneration [26]; therefore timing of therapy may be crucial.

Using Fcγ receptor expressing NK cells, it has been shown that anti-C1q antibody does not attenuate antibody-dependent cytotoxicity [28]. This suggests that any attenuation of injury reported here and in the NMO spinal cord demyelinating injury model most likely has been through mediation of the complement pathway [28]. Our model currently concentrates on one aspect of AMAN pathogenesis and its inhibition; namely anti-ganglioside antibody activation of the classical complement pathway and subsequent complement-dependent nerve injury and its attenuation by anti-C1q antibody, respectively. However, anti-ganglioside antibody can conceivably cause injury through complement-independent mechanisms. Indeed, it has recently been reported that nodal and axonal injury can be mediated through anti-ganglioside antibody immune complex formation and recruitment of macrophages through their Fcγ receptors in a mouse model [16]. It is likely that more than one mechanism will be active simultaneously in human GBS, and it is therefore important to broadly consider inflammatory pathways whilst implementing therapies.

The original characterisation of anti-C1q monoclonal antibody demonstrated an apparent binding affinity of 11 pM, which is comparable to other approved monoclonal antibody therapeutics including rituximab to its antigen CD20 (~8nM) [28, 33]. The results provided here suggest that C1q is a valid target for GBS, and the next step will be the generation of a humanized, non-immunogenic antibody with suitable pharmacokinetics in humans.
